# Evaluation of Electro-Neuro-Feedback (ENF) Treatment in Reducing Post-Surgical Hematoma in Young and Active Patients Undergoing Total Hip Replacement

**DOI:** 10.3390/medicina62071264

**Published:** 2026-06-30

**Authors:** Cristina Beretta, Cesare Donarini, Claudio Legnani, Filippo Saluzzo

**Affiliations:** 1 Department of Orthopaedic Rehabilitation, IRCCS Istituto Ortopedico Galeazzi, 20148 Milan, Italy; 2 Sport Traumatology and Minimally Invasive Surgery Center, IRCCS Istituto Ortopedico Galeazzi, 20148 Milan, Italy

**Keywords:** total hip replacement, total hip arthroplasty, post-operative hematoma, electro-neuro-feedback, ENF

## Abstract

*Background and Objectives*: Postoperative hematomas are relatively uncommon complications following total hip replacement (THR) which could result in persistent pain, surgical site infections, neurological deficits, and may require reoperation. The aim of this study is to evaluate the impact of electro-neuro-feedback (ENF), a portable Transcutaneous Electric Nerve Stimulation, in the management of hematomas following THR, focusing on postoperative pain, clinical outcome, and recovery time in a cohort of relatively young and active patients. *Materials and Methods*: Five 10-minute cycles of ENF Physio of risk class IIa, certificated 0476 were applied. Outcomes included hematoma volume (measured with triaxial ultrasound), thigh circumference, Visual Analog Scale (VAS), Global Perceived Effect (GPE) scale, and HOOS-Physical Function Short (HOOS-PS) form. *Results*: Twenty participants were enrolled. The reduction in hematoma volume was statistically significant (*p*-value < 0.001) after five ENF sessions from a pre-treatment mean value of 337.2 mL (SD: 160.5) to 115.9 mL (SD: 78.1) at follow-up. Each patient had a reduction in the hematoma volume superior to 20%. No difference in thigh circumference before and after treatment was reported (*p*-value = 0.694). Pain according to VAS significantly decreased at follow-up (*p* < 0.05). *Conclusions*: The application of ENF was associated with reduced hematoma volume and post-operative pain. These findings can pave the way for future research to evaluate the risks of hematoma following THR.

## 1. Introduction

Total hip replacement (THR) is an effective procedure in patients with end-stage hip osteoarthritis [1]. In Italy, elective THRs increased by 44.5% from 2011 (39,144) to 2014 (56,561), with a steady annual growth rate of 2.9% [2]. Although traditionally reserved for the elderly population, this surgical procedure is increasingly being used to treat OA in younger patients—a group with higher functional demands in terms of a quick return to work and recreational activities [3,4].

Among complications following hip surgery, hematomas can develop from skin and muscle trauma related to surgery, inadequate hemostasis, and therapeutic anticoagulation [5,6,7]. Although hematomas following THR are relatively uncommon, occurring in about 0.41% to 1.7% of surgical procedures [5,8], they can increase the risk of persistent pain, neurological impairment, surgical site infections, and may require reoperation [8,9].

Transcutaneous Electrical Nerve Stimulation (TENS) serves as a valuable, non-pharmacological adjunct in peri-operative analgesia [10,11]. TENS devices utilize electric impulses delivered to tissues. These devices generate waveforms that automatically adapt to real-time variations in skin impedance through a feedback compensation algorithm. Their action promotes tissue regeneration, while facilitating the gate control mechanism of pain modulation. Electro-Neuro-Feedback (ENF) is a portable TENS device. The clinical use of ENF (and TENS, in general) has achieved favorable results in medical fields outside of orthopedics and physical therapy, and its role in reducing postsurgical swelling and local edema has been reported in oral surgery [12]. However, there is growing interest in applying this type of physiotherapeutic treatment to reduce post-surgery pain and allow faster rehabilitation, especially in the early post-operative period [13].

The purpose of this study was to report on the results of ENF treatment in the reabsorption of hematomas following THR and on postoperative pain, aiming to allow a faster return to work and sports in young, active populations. Additional endpoints of the study were to evaluate patient-related outcomes and recovery time.

## 2. Materials and Methods

### 2.1. Study Design

A prospective, single-center, observational cohort study was conducted at the Department of Orthopaedic Rehabilitation of our Institute. The study has been approved by the Ethic Committee of the San Raffaele Hospital, Milan Italy on 14 July 2021 (IRB Approval Number 122/INT/2021) and has been conducted in accordance with the principles set forth in the Helsinki Declaration [14]. This study was conducted according to the STROBE guidelines for observational studies [15] and linked to a ClinicalTrials.gov identifier (Clinical trial registry number NCT05196321). Patients signed written informed consent.

### 2.2. Patients’ Population

Twenty consecutive patients who underwent THR and developed post-surgical hematoma for whom the use of an ENF device was planned were prospectively enrolled between November 2021 and October 2023. Inclusion criteria were as follows: (1) age ≥ 18 and <60 years old; (2) Lower Extremity Activity Scale (LEAS) score greater than or equal to 10; (3) suitable for the use of the ENF device (4) follow-up duration at least five days following THR surgery (5) no history of neurological or psychiatric pathologies. Patients with severe cardiac conditions, pacemakers or medullary stimulators, or active cancers in the treatment area, as well as those undergoing revision THR or bilateral hip replacement, were excluded.

### 2.3. Surgical Technique and Rehabilitation Protocol

THR was performed by the same surgical team using a posterolateral tissue sparing technique. Postoperatively, patients were administered non-steroidal anti-inflammatory drugs (NSAIDs) and analgesics, in accordance with the institute’s protocol. Post-operative rehabilitation included mobilization and walking with crutches with partial weight-bearing starting from the first postoperative day.

### 2.4. ENF Equipment and Treatment Protocol

The present study utilized the ENF Physio (Fast Therapies, Carpenedolo, Italy). Electrical impulses had a duration of 3.3–483 ms. The device’s current dynamically adapted to tissue response and local impedance through the modulation of electrical parameters: frequency ranged from 15.3 to 354 Hz, and voltage from 320 to 450 V. The stimulation frequency was initially set at 80 Hz, and gradually increased to 120 Hz, or until patients reported a tingling sensation, adjusted to each patient’s highest tolerable stimulation level. Sessions lasted 10 min and were administered daily in the afternoon during rehabilitation. The protocol featured two consecutive 5-minute phases (acute anti-inflammatory and deep draining), both administered using the brush technique. The brush technique involves the continuous and controlled sliding of the active electrode across the patient’s skin surface in the area surrounding the hematoma close to the surgical dressing. No additional physical therapies were performed during ENF treatment. The protocol was standardized for all patients. A single trained operator performed all procedures (F.S.).

### 2.5. Outcome Measures

The primary outcome was the change in size (volume reduction) of the post-surgical hematoma, measured via triaxial ultrasound at the end of treatment on the 5th postoperative day. Ultrasound examinations were performed with a MyLab 25 ultrasound system (Esaote, Genoa, Italy) using a linear probe (7.5 MHz). A hematoma was defined as an echogenic area surrounding the incision site. The maximum thickness of each hematoma was recorded in millimeters, and the hematoma volume was calculated using the spheroid formula (length × width × depth × π/6). Examinations were performed with patients in both the supine and lateral positions. The ultrasound scans were conducted by a physician (C.D.) experienced in musculoskeletal radiology.

The secondary outcomes included changes in thigh circumference, as well as pain intensity quantified by the VAS (0–10), both measured before and after treatment. The GPE scale was used to assess perceived global recovery [16], and the HOOS-PS scale was used to evaluate patient satisfaction at follow-up [17,18]. Adverse events were recorded throughout the duration of the study.

### 2.6. Statistical Analysis

The continuous variables are expressed as mean ± standard deviation (SD) and median (interquartile range), and the categorial variables are expressed as absolute frequency. The analysis of the variables of hematoma volume, thigh circumference, and VAS was performed by comparing the pre- and post-treatment. The analysis of the data distribution was conducted through the Shapiro–Wilk test. The variables with a normal distribution were analyzed by the paired Student *t* tests. In case of non-normal distribution, the Wilcoxon matched-pairs signed rank test was used.

The analysis was conducted on GraphPad Prism 8.3.0 (GraphPad Software Inc., San Diego, CA, USA). The results were considered statistically significant for *p*-values < 0.05.

We estimated a reduction of at least 20% of the post-surgery hematoma as the primary outcome and a standard deviation of the sample of 20%; then, the sample size for the Student *t*-test for repeated measures with a significance level of 0.05 and power of 95% was 16 participants. By adding 20% of participants for possible dropout, we determined a sample size of 20 patients.

## 3. Results

Of the 20 patients enrolled, 18 (90%) were male, and two (10%) were female. Mean age at time of surgery was 53.0 years (SD: 5.4). Table 1 reports data relative to the descriptive analysis of our patient population: demographic characteristics of the patients and the pre-treatment values of hematoma volume, thigh circumference, and VAS values.

None of the patients examined reported adverse events during the perioperative period.

Average hematoma volume significantly decreased after five ENF sessions compared to pre-treatment status (*p* < 0.001, Table 2), from a mean value of 337.2 mL (SD: 160.5) to 115.9 mL (SD: 78.1). Reduced VAS scores were reported after five ENF sessions compared to pre-treatment (*p* < 0.05, Table 3).

The difference was observed for each of the 20 patients enrolled in the study, with a reduction in the hematoma volume superior to 30% in every patient (Table 3).

Concerning patient-reported outcome scores, no differences were reported concerning HOOS-PS score, and GPE at follow-up (p: n.s., Table 4).

## 4. Discussion

The application of ENF was associated with a reduction in hematoma volume and post-operative pain in young, active patients who developed post-surgical hematomas after THR. Overall, an average decrease in hematoma volume of more than 60% was observed after five sessions of ENF, as assessed by ultrasound. Regarding pain, a significant reduction in the VAS score was noted at follow-up compared to the pre-treatment status, achieving a 29% mean reduction. These improvements were corroborated by patient-reported outcomes on the HOOS-PS and GPE scales, both of which indicated good-to-excellent results.

Limited research exists on the application of TENS in the postsurgical phase; however, available studies primarily evaluate pain reduction using the Visual Analog Scale (VAS) or Numerical Rating Scale (NRS). Furthermore, a meta-analysis of five randomized controlled trials involving 472 total knee arthroplasty patients revealed that TENS significantly diminishes both pain and opioid consumption [11].

In a double-blind, randomized trial involving 41 elderly patients (mean age: 78–80 years), Elboim-Gabyzon et al. evaluated the effects of adding continuous transcutaneous electrical nerve stimulation (1000 Hz frequency, 200 ms phase duration) to standard rehabilitation care. The study focused on patients in the acute post-operative phase following Gamma-nail fixation of extracapsular hip fractures. Between the second and fifth post-operative days, a statistically significant advantage in pain reduction and mobility—as measured by the Functional Ambulation Classification instrument—was observed compared to the control group (*p* < 0.0011) [19].

Similar positive results have been achieved by Oksar et al., who assessed the reduction in post-operative acute pain after hip fracture surgery in a double-blinded, randomized clinical trial that involved 120 patients (ASA class I or II) [20]. The investigators compared three groups of patients: (a) those who received patient-controlled analgesia (PCA) with epidural fentanyl and additional medications; (b) those who received lumbar plexus and sciatic nerve transcutaneous electrical nerve stimulation (LS-TENS) alongside the same medical protocol; and (c) those who received surgical wound transcutaneous electrical nerve stimulation (SW-TENS) alongside the same medical protocol. VAS scores were higher in the control group than in the SW-TENS group at 30 min (*p* < 0.05) and in the SW-TENS group than in the LS-TENS group at 24 h post-surgery (*p* < 0.05). Furthermore, the 48 h total level of analgesic consumption was higher in the control group than in the LS-TENS group (*p* < 0.05) [20]. Similarly, Opolka et al. evaluated mixed-frequency TENS (2 Hz/80 Hz) as an adjunct to standard care during the first 2.5 h post-hip surgery (following hip fractures or elective THR) in a single-blinded, randomized, placebo-controlled trial. The study involved 30 patients aged over 18 years [21]. TENS was applied in two 30-minute sessions. To simplify the application, TENS was administered using specific pants with integrated modular textile electrodes that allow for stimulation at rest and during activity. Patients in the active TENS group exhibited significantly higher Patient Global Impression of Change scores at 30 min, which were sustained throughout the intervention (*p* ≤ 0.001), compared to the control group. Self-reported pain ratings (NRS) were reduced after one hour of active TENS, and this reduction continued throughout the intervention (*p* ≤ 0.005). No side effects or postoperative complications were detected during the study, and no significant difference was observed between groups regarding opioid consumption.

Major wound complications, which include hematomas, are among the most frequent post-surgery complications of THR, and several studies investigated have investigated possible differences among surgical techniques. For example, in a high-volume Ontario tertiary care center, the direct anterior approach was linked to major wound complications reported in 28 out of 875 (3.2%) patients within one year of THR [1]. Of these 28 cases, hematoma accounted for five incidents (17.8%). Additionally, a higher BMI was significantly associated with an increased risk of wound complications.

Study limitations include the relatively low number of patients, the short follow-up period, and the lack of a control group. The limited study population may not have allowed for detection of small differences between pre-treatment and follow-up regarding some parameters. Although a trend was noted in the reduction in thigh circumference, significancy was not observed. A greater number of patients could have enhanced the power of the results obtained. However, this may have been complicated in a prospective study design on a relatively infrequent complication in a selected cohort of young and active patients. GPE and HOOS-PS results were presented only at follow-up without baseline values, thereby limiting the assessment of clinical improvement. Additional study limitations include potential spontaneous hematoma resorption, as well as possible placebo or nonspecific effects of extra clinical attention. Finally, incorporating longer-term outcomes—such as return to function, infection rates, reoperation, or hematoma persistence—would have enhanced the validity of the findings. Given these limitations, these results should be considered preliminary and require confirmation. Further randomized controlled trials with larger cohorts are needed to confirm the efficacy and validity of the ENF procedure in managing postoperative hematomas following THR.

## 5. Conclusions

The application of ENF was associated with decreased hematoma volume, as measured by ultrasound, in relatively young and active patients who experienced post-surgical hematomas following THR. Furthermore, VAS scores decreased significantly at follow-up compared to baseline. Good to excellent patient-reported outcomes scores were reported after treatment.

## Figures and Tables

**Table 1:** Descriptive analysis of the patients’ population before treatment.

Age (ys)	
mean (SD)	53.0 (5.4)
median (range)	53.0 (49.2–55.0)
Sex	
male	18
female	2
Hematoma volume (mL)	
mean (SD)	337.2 (160.5)
median (range)	410.8 (55.1–562.3)
Thigh circumference (cm)	
mean (SD)	68.5 (7.2)
median (range)	70.0 (63.0–73.7)
VAS	
mean (SD)	4.5 (1.8)
median (range)	5.0 (3.0–6.0)

SD: standard deviation, VAS: Visual Analog Scale.

**Table 2:** Analysis of hematoma volume, thigh circumference, and VAS before and after treatment.

	Pre-Treatment	Follow-Up	*p*-Value
Hematoma volume (mL)			
mean (SD)	337.2 (160.5)	115.9 (78.1)	<0.001
median (range)	410.8 (55.1–562.3)	124.7 (4.3–295.3)	
Thigh circumference (cm)			
mean (SD)	68.5 (7.2)	66.7 (11.9)	0.694
median (range)	70.0 (63.0–73.7)	69.5 (61.9–74.7)	
VAS			
Mean (SD)	4.5 (1.8)	3.1 (1.6)	0.022
median (range)	5.0 (3.0–6.0)	3.0 (2.0–5.0)	

VAS: Visual Analog Scale, SD: standard deviation.

**Table 3:** Reduction in hematoma volume (mL, percentage) at the beginning of the study and after five sessions of ENF (follow-up) per each patient enrolled in the study.

Patients	Hematoma Volume Pre-Treatment (mL)	Hematoma Volume at Follow-Up (mL)	% Hematoma Reduction
1	238.6	161.8	−32.2
2	107.6	10.4	−90.3
3	55.1	4.3	−93.3
4	112.8	14.1	−87.5
5	146.2	8.9	−93.1
6	202.8	58.4	−71.2
7	108.8	47.1	−56.7
8	474.9	127.6	−73.1
9	465.2	130.0	−72.1
10	408.8	235.8	−42.3
11	458.6	124.3	−72.9
12	562.3	93.4	−83.4
13	359.6	92.2	−74.4
14	453.1	156.8	−65.4
15	449.5	118.2	−73.7
16	469.7	295.3	−37.1
17	479.7	125.1	−73.7
18	455.8	145.4	−68.1
19	412.9	183.9	−55.5
20	321.6	185.2	−42.4

Similarly, no difference in thigh circumference was detected pre-treatment and at follow-up (*p*-value = 0.694, Table 3).

**Table 4:** HOOS-PS, GPE, adverse events measured after the fifth session of ENF.

	Follow-Up
HOOS-PS	
mean (SD)	80.1 (13.1)
median (range)	80.5 (69.5–91.0)
GPE	
A	13
B	7
Adverse events	0

## Data Availability

Data are available upon reasonable request.

## Abbreviations

The following abbreviations are used in this manuscript:

THR	Total Hip Replacement
TENS	Transcutaneous Electric Nerve Stimulation
ENF	Electro-Neuro-Feedback
VAS	Visual Analog Scale
GPE	Global Perceived Effect
HOOS-PS	HOOS-Physical Function Short
